# Stat3 mediates Fyn kinase-driven dopaminergic neurodegeneration and microglia activation

**DOI:** 10.1242/dmm.052011

**Published:** 2024-12-06

**Authors:** Sahiba Siddiqui, Fang Liu, Anumantha G. Kanthasamy, Maura McGrail

**Affiliations:** ^1^ Department of Genetics, Development, and Cell Biology, Iowa State University, Ames, IA 50011, USA; ^2^ Interdepartmental Genetics and Genomics Graduate Program (IGG), Iowa State University, Ames, IA 50011, USA; ^3^ Center for Brain Science and Neurodegenerative Diseases, Department of Physiology and Pharmacology, University of Georgia, Athens, GA 30602, USA

**Keywords:** Zebrafish neurodegeneration model, Fyn kinase, Dopaminergic neuron, Microglia activation, Mitophagy, STAT3, TNF-α, NF-κB

## Abstract

The Alzheimer's disease and Parkinson's disease risk locus FYN kinase is implicated in neurodegeneration and inflammatory signaling. To investigate *in vivo* mechanisms of Fyn-driven neurodegeneration, we built a zebrafish neural-specific Gal4:UAS model of constitutively active FynY531F signaling. Using *in vivo* live imaging, we demonstrated that neural FynY531F expression leads to dopaminergic neuron loss and mitochondrial aggregation in 5 day larval brain. Dopaminergic loss coincided with microglia activation and induction of *tnfa*, *il1b* and *il12a* inflammatory cytokine expression. Transcriptome analysis revealed Stat3 signaling as a potential Fyn target. Chemical inhibition experiments confirmed Fyn-driven dopaminergic neuron loss, and the inflammatory response was dependent upon activation of Stat3 and NF-κB pathways. Dual chemical inhibition demonstrated that Stat3 acts synergistically with NF-κB in dopaminergic neuron degeneration. These results identify Stat3 as a novel downstream effector of Fyn signaling in neurodegeneration and inflammation.

## Teaser

**Summary:** This study describes a novel *in vivo* zebrafish model of neural Fyn kinase activation and identifies Stat3 signaling as a downstream Fyn effector in dopaminergic neuron degeneration and neuroinflammation.

## INTRODUCTION

Cellular kinases play a central role in protein aggregation in dementia and neurodegeneration, and their involvement in inflammatory signaling in Alzheimer's disease (AD) and related diseases such as Parkinson's disease (PD) is well established. The SRC family member FYN kinase has a key role in multiple neurodegenerative disorders (Guglietti et al., 2021). FYN has been implicated in AD amyloid-β, tau and PD α-synuclein protein aggregate signaling (Nygaard, 2018), and has been identified as a genome-wide association study PD risk locus (Nalls et al., 2019). Increased FYN expression and phosphorylation, a marker of FYN activation, has been identified in AD and PD patient brain tissue (Guglietti et al., 2024; Low et al., 2021; Panicker et al., 2019), and FYN activation has been reported to correlate with microglia activation (Panicker et al., 2019)*. In vitro* analyses of FYN function indicate a role for FYN signaling in dopaminergic neurons and microglia activation in neurodegeneration. FYN was shown to activate PKC-δ (also known as PRKCD) phosphorylation and oxidative stress-induced cell death in rat dopaminergic neurons (Kaul et al., 2005; Saminathan et al., 2011). In microglia, FYN signaling stimulated inflammasome activation and cytokine induction through the PKC-δ/NF-κB pathway (Panicker et al., 2015, 2019). In primary microglia, FYN leads to upregulation and phosphorylation of the Kv1.3 voltage-gated calcium channel (also known as KCNA3), presenting a second pathway of FYN-driven microglia activation in neuroinflammation (Sarkar et al., 2020). FYN knockout mice demonstrate a requirement for FYN in neurotoxin-induced dopaminergic neuron loss and microglia inflammatory response (Panicker et al., 2015). However, the mechanism by which elevated FYN signaling drives neurodegeneration *in vivo* has not been fully investigated.

The zebrafish provides a powerful *in vivo* platform for modeling neurodegeneration (Chia et al., 2022). Neurotoxin and genetic zebrafish models of PD risk loci indicate that conserved mechanisms underlie dopaminergic neurodegeneration (Flinn et al., 2009, 2013; Godoy et al., 2015; Kalyn et al., 2019; Omar et al., 2023). Dopaminergic neurons in larval and adult zebrafish forebrain are well described (Holzschuh et al., 2001; Kaslin and Panula, 2001; Wullimann and Rink, 2001), and A11-related dopaminergic neurons controlling mechanosensation, locomotion and vision have been identified (Löhr et al., 2009; Reinig et al., 2017; Tay et al., 2011). Dopaminergic ventral diencephalon (vDC) clusters located in the posterior tuberculum of the vDC until recently were considered analogous to the human dopaminergic neurons in the substantia nigra pars compacta (SNc) of the midbrain (Xi et al., 2011b), which are lost in neurological diseases resulting in movement disorders such as PD. More recently, Th-positive neurons have been identified in the midbrain and in the hindbrain adjacent to the midbrain–hindbrain border, suggesting potential additional populations of dopaminergic neurons analogous to the human midbrain SNc controlling locomotion (Altburger et al., 2023). Zebrafish transgenic reporter lines *dat:eGFP* (Xi et al., 2011b) and *dat:mitoRFP* (Noble et al., 2015) for *in vivo* live imaging of dopaminergic neurons and mitochondria in the larval brain provide powerful tools for analysis of chemical and genetic models of dopaminergic neuron degeneration (Godoy et al., 2015; Kalyn et al., 2019; Xi et al., 2011a).

Using the binary Gal4; UAS system for cell type-specific gene expression, we created an *in vivo* model of activated Fyn (also known as Fyna in zebrafish) kinase signaling that drives neurodegeneration to investigate mechanisms of Fyn-driven neurodegeneration. Neural-specific expression of the constitutively active Fyn mutant Y531F led to larval morphological and phenotypic defects that recapitulate previously described zebrafish neurodegeneration models. Live imaging of larval brains revealed that Fyn signaling drove the loss of *dat:eGFP*-labeled vDC dopaminergic cell clusters and cell body aggregation of *dat:mitoRFP*-labeled mitochondria over the course of 3 days post fertilization (dpf) to 5 dpf. Dopaminergic neuron loss correlated with a shift in microglia morphology from ramified to amoeboid, consistent with elevated expression of the microglial activation marker *acod1*/*irg1* and induction of inflammatory cytokine expression. Transcriptome analysis revealed alterations indicative of disrupted mitochondrial oxidative phosphorylation and metabolic pathways, and revealed Stat3 as a novel Fyn downstream effector. Chemical inhibition indicated that dopaminergic neuron loss and inflammatory gene expression were dependent on both Stat3 and NF-κB pathways. Together, Stat3 and NF-κB were shown to synergize with Fyn-activated signaling in dopaminergic neuron loss. Our model of neural Fyn kinase signaling provides a new *in vivo* platform for investigating Stat3 targets and mechanisms of organelle stress in Fyn-driven dopaminergic neurodegeneration.

## RESULTS

### Zebrafish model of Fyn kinase-driven neurodegeneration

**Fig. 1.**
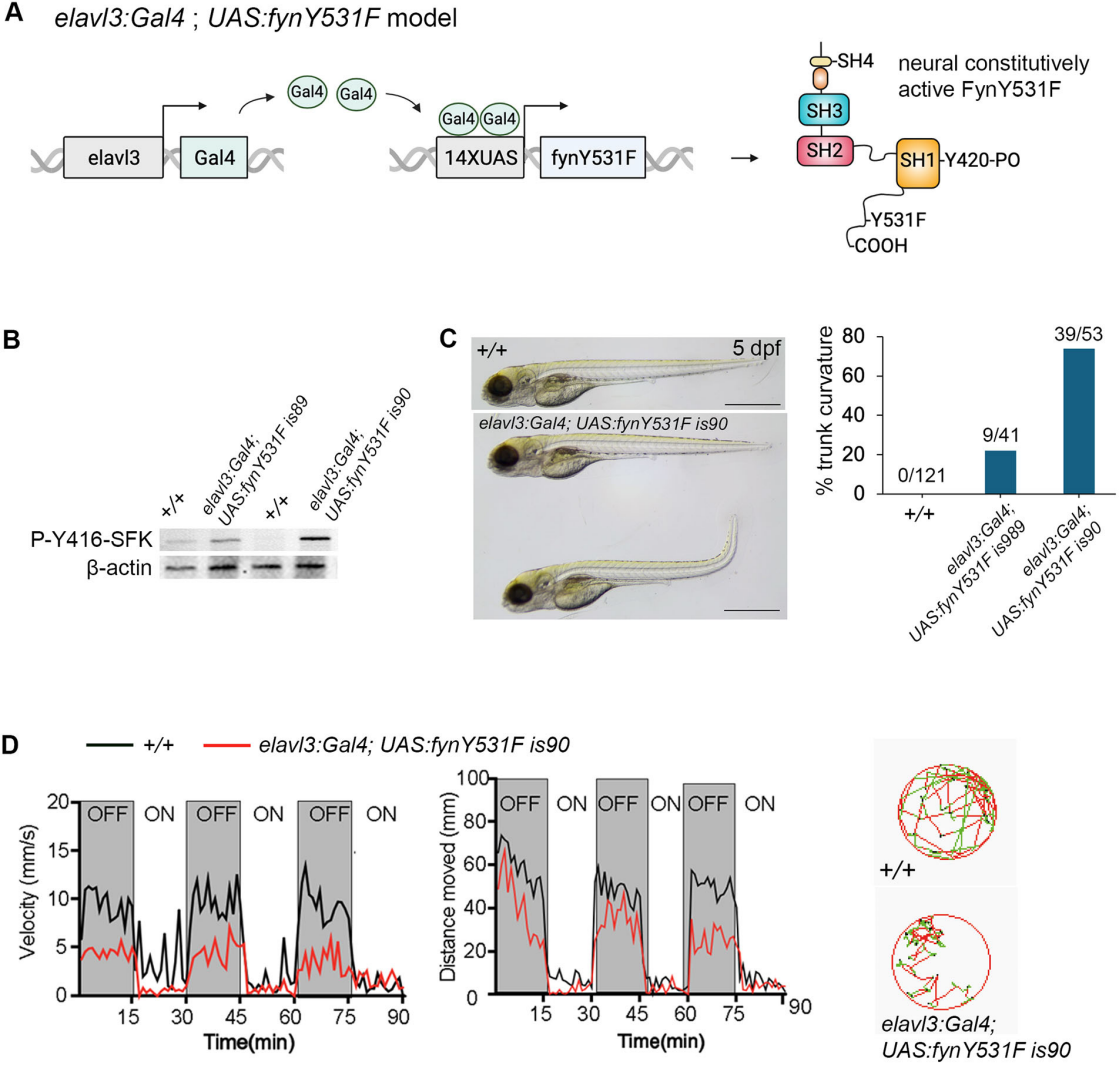
**Gal4-UAS overexpression of FynY531F leads to autophosphorylation of Y416, trunk curvature and swimming defects.** (A) Diagram of *elavl3:Gal4; 14XUAS:fynY531F* neural-specific model. (B) Western blot probed with anti-P-Y416-SFK antibody, showing elevated Y416 phosphorylation in 5 days post fertilization (dpf) *elavl3:Gal4; 14XUAS:fynY531Fis89* and *elavl3:Gal4; 14XUAS:fynY531Fis90* in comparison to wild-type +/+ larvae. Anti-β-actin antibody was used as a loading control. (C) Trunk curvature observed in a subset of 5 dpf *elavl3:Gal4; 14XUAS:fynY531Fis90* larvae. Plot shows the percentages of 5 dpf wild-type +/+, *elavl3:Gal4; 14XUAS:fynY531Fis89* and *elavl3:Gal4; 14XUAS:fynY531Fis90* larvae showing trunk curvature. (D) Measurements of velocity (left) and distance moved (middle) in 5 dpf wild-type +/+ and *elavl3:Gal4; 14XUAS:fynY531Fis90* larvae that showed normal trunk morphology. Circular plots (right) show 5 s snapshots of tracking movements. Normal movements (green lines) and large movements (red lines) are shown for a wild-type 5 dpf +/+ larva and an *elavl3:Gal4; 14XUAS:fynY531Fis90* larva with normal trunk morphology. Scale bars: 500 µm.

To build a zebrafish model of Fyn-mediated neurodegeneration, we used the two-component Gal4-UAS system to drive overexpression of the constitutively active mutant FynY531F in neurons (Fig. 1). The zebrafish *fyna* Y531 residue is analogous to human Y528 located in the regulatory C-terminal domain of the protein. The Y528F phenylalanine phospho-null substitution mutation prevents the FYN C-terminal tail from inhibiting the active site of the SH1 kinase domain, leading to a constitutively active kinase that drives persistent production of inflammatory cytokine interleukin 1β (IL-1β) (Takeuchi et al., 1993). The zebrafish *fynaY531F* mutant cDNA was cloned into the *pTol2<14XUAS; gcry1:mRFP>* vector (Balciuniene et al., 2013), and two independent transgenic lines were established by standard Tol2 insertional transgenesis (Balciunas et al., 2006). The *fynaY531F* transgenic lines used in this study are *Tol2(14XUAS:fynaY531F; gcry1:mRFP)^is89^* (*UAS:fynY53F1is89*) and *Tol2(14XUAS:fynaY531F; gcry1:mRFP)^is90^* (*UAS:fynY531Fis90*).

To determine whether neural expression of constitutively active FynY531F would lead to neurodegeneration, we crossed the *UAS:fynY531F* lines with a neural-specific Gal4 driver, *Tg(elavl3:Gal4=VP16)^nns6^* (Kimura et al., 2008) (designated as *elavl3:Gal4*) (Fig. 1A). Western blots of extracts from 5 dpf *elavl3:Gal4;UAS:fynY531F* larvae were probed with an antibody that recognizes the autophosphorylation of tyrosine residue Y416 in the SH1 kinase domain of Src family kinases (p-Y416-SFK), corresponding to Y420 in Fyn kinase (Kouadir et al., 2012; Um et al., 2012; Wake et al., 2011). Neural-specific expression of Y531F Fyn led to increased levels of p-Y416-SFK in comparison to those in wild-type larvae (Fig. 1B), consistent with elevated Fyn kinase phosphorylation activity.

Neural expression of FynY531F resulted in 22% of 5 dpf *elavl3:Gal4; UAS:fynY5341Fis89* and 74% of 5 dpf *elavl3:Gal4; UAS:fynY531is90* larvae showing a curved trunk (Fig. 1C). To determine the impact of FynY531F overexpression on locomotory behavior, *elavl3:Gal4; UAS:fynY531Fis90* larvae with normal morphology were selected. The velocity and distance of light-induced swimming over time was measured following previously established parameters (de Esch et al., 2012). Compared to wild-type larvae, the *elavl3:Gal4; UAS:fynY531Fis90* larvae showed a reduction in both swimming velocity and distance (Fig. 1D). These results indicated that elevated levels of Fyn signaling in the *elavl3:Gal4; UAS:fynY531F* model correlate with morphological and motor defects that mimic previously described zebrafish models of neurodegeneration induced by the neurotoxin 1-methyl-4-phenyl-1,2,3,6-tetrahydropyridine (MPTP) (Godoy et al., 2015; Kalyn et al., 2019; McKinley et al., 2005).

In the experiments outlined in this study, line *UAS:fynY531Fis90* was crossed to the dopamine transporter (*dat*; also known as *slc6a3*) GFP reporter *Tg(dat:eGFP)* (Xi et al., 2011b), and double transgenic adult *dat:eGFP; UAS:fynY531Fis90* were maintained and used to generate control and *elavl3:Gal4; 531F* model larvae for all confocal live-imaging experiments. Line *UAS:fynY5341Fis89* was used to generate control, and *elavl3:Gal4; fynY531F* was used for all other experiments.

### Activated Fyn signaling leads to dopaminergic neuron loss and mitochondria accumulation

**Fig. 2.**
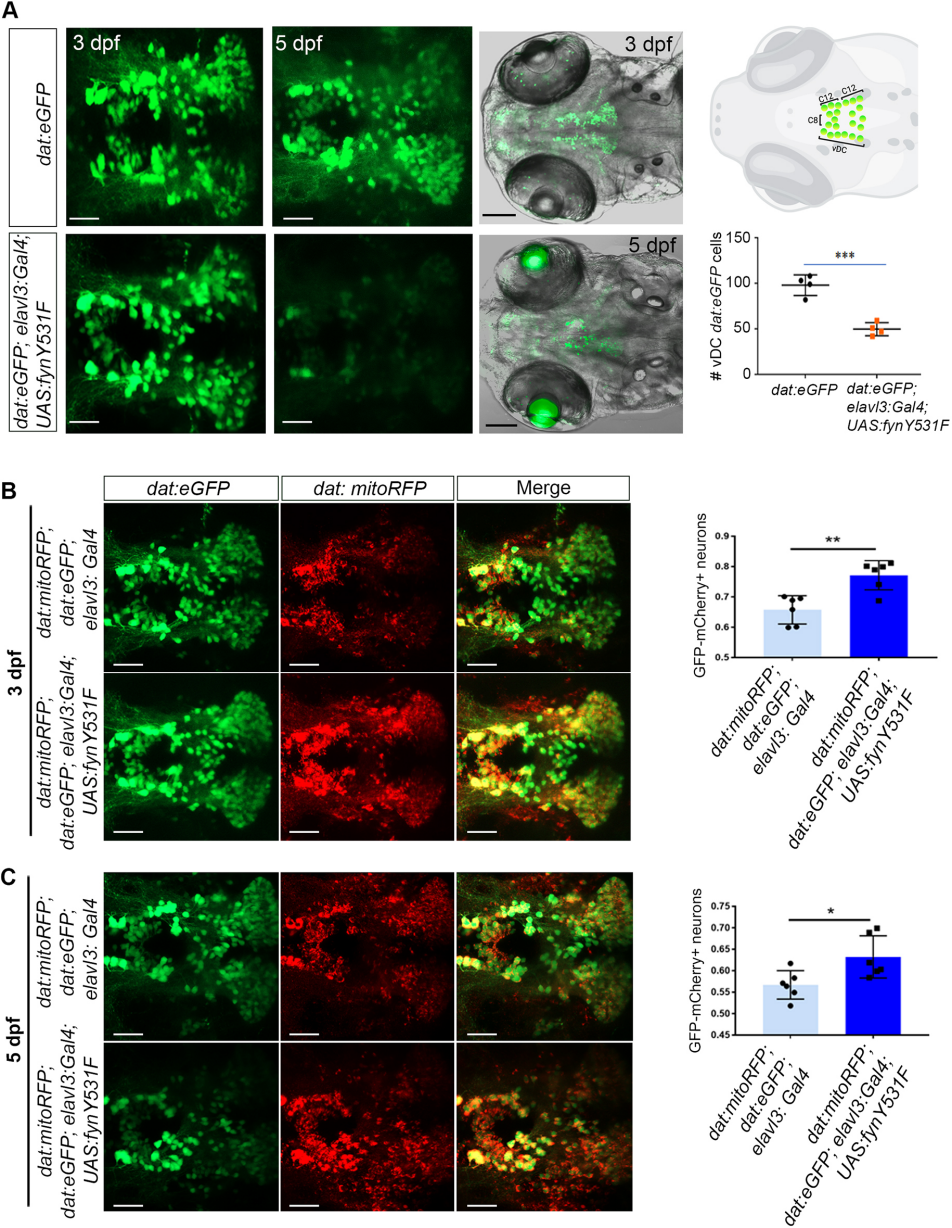
**Zebrafish neural FynY531F overexpression drives dopaminergic neuron loss and mitochondria accumulation.** (A) Left: live confocal imaging of ventral diencephalon (vDC) neuron cluster eGFP dopaminergic neurons in 3 dpf and 5 dpf control *dat:eGFP* and *elavl3:Gal4; UAS:fynY531F* larvae. Top right: vDC in 5 dpf larval brain. Diagram adapted from Kalyn et al. (2019) and created with BioRender.com. Bottom right: quantification of vDC eGFP neuronal cell bodies in 5 dpf control *dat:eGFP* and *dat:eGFP; elavl3:Gal4; UAS:fynY531F* larvae (*n*=4). (B) Left: live confocal imaging of eGFP and mCherry in vDC neurons in 3 dpf control *dat:mitoRFP*; *dat:eGFP*; *elavl3:Gal4* and *dat:mitoRFP*; *dat:eGFP*; *elavl3:Gal4; UAS:fynY531F* larval brain. Right: quantification of overlap of eGFP and mCherry signal in vDC neuron cell bodies in 3 dpf control *dat:mitoRFP*; *dat:eGFP*; *elavl3:Gal4* and *dat:mitoRFP*; *dat:eGFP*; *elavl3:Gal4; UAS:fynY531F* larvae (*n*=6). (C) Left: live confocal imaging of eGFP and mCherry in vDC neurons in 5 dpf control *dat:mitoRFP*; *dat:eGFP*; *elavl3:Gal4* and *dat:mitoRFP*; *dat:eGFP*; *elavl3:Gal4; UAS:fynY531F* larval brain. Right: quantification of overlap of eGFP and mCherry signal in vDC neuron cell bodies in 5 dpf control *dat:mitoRFP*; *dat:eGFP*; *elavl3:Gal4* and *dat:mitoRFP*; *dat:eGFP*; *elavl3:Gal4; UAS:fynY531F* larvae (*n*=6). Statistical analysis was performed with two-tailed unpaired Student’s *t*-test. Bars represent mean±s.e.m. *P<0.05; **P<0.01; ***P<0.001. Scale bars: 50 µm (white); 100 µm (black).

To determine whether the zebrafish neural FynY531F model led to neurodegeneration, live confocal imaging of dopaminergic neurons was performed using *Tg(dat:eGFP)* (Xi et al., 2011b) (Fig. 2). Compared to wild-type *dat:eGFP* control larvae, at 3 dpf, *dat:eGFP; elavl3:Gal4; UAS:fynY531F* larvae did not show a significant difference in the number of eGFP-positive cells in the vDC clusters of dopaminergic neurons in the forebrain (*P*=0.0937) (Fig. 2A). In 5 dpf *dat:eGFP; elavl3:Gal4; UAS:fynY531F* larvae, the level of dat:eGFP signal was reduced overall in the forebrain, midbrain, cerebellum and hindbrain, and the number of eGFP-positive cells in the vDC clusters was significantly reduced compared to that in the control (*P*=0.0004) (Fig. 2A). These results indicated that activated FynY531F signaling leads to dopaminergic neuron loss, similar to the loss described in *pink* (also known as *pink1*) genetic (Flinn et al., 2013), *parkin* (also known as *prkn*) morpholino knockdown (Flinn et al., 2009) and MPTP neurotoxin (Godoy et al., 2015; Kalyn et al., 2019; McKinley et al., 2005; Xi et al., 2011b) zebrafish models of neurodegeneration.

A hallmark of neurodegeneration is loss of mitochondria (Klemmensen et al., 2024). To examine whether activated Fyn signaling impacts mitochondria in dopaminergic neurons, live confocal imaging of the dopamine mitochondrial fluorescent mCherry reporter line *Tg(dat:tom20 MLS-mCherry)* (*dat:mitoRFP*) (Noble et al., 2015) was performed in control *elavl3:Gal4* and *elavl3:Gal4; UAS:fynY531Fis90* larvae at 3 dpf (Fig. 2B) and 5 dpf (Fig. 2C). We observed that 3 dpf *dat:eGFP*; *dat:mitoRFP; elavl3:Gal4; UAS:fynY531Fis90* larvae showed an increase in mCherry signal colocalizing with eGFP in dopaminergic vDC cell bodies compared to that in control larvae (*P*=0.0019) (Fig. 2B). At 5 dpf, colocalization of mCherry signal with eGFP in remaining vDC cell bodies was significantly higher than that in controls (*P*=0.0228) (Fig. 2C), despite the reduction in overall vDC number in *dat:eGFP; dat:mitoRFP; elavl3:Gal4; UAS:fynY531Fis90* larvae. The increase in *dat:mitoRFP* signal suggests that mitochondria accumulate in the dopaminergic cell bodies of *elavl3:Gal4; UAS:fynY531Fis90* larvae, suggesting a possible role for mitochondrial dysfunction in dopaminergic neuron loss.

### Activated Fyn leads to induction of inflammatory cytokines and microglia activation

**Fig. 3.**
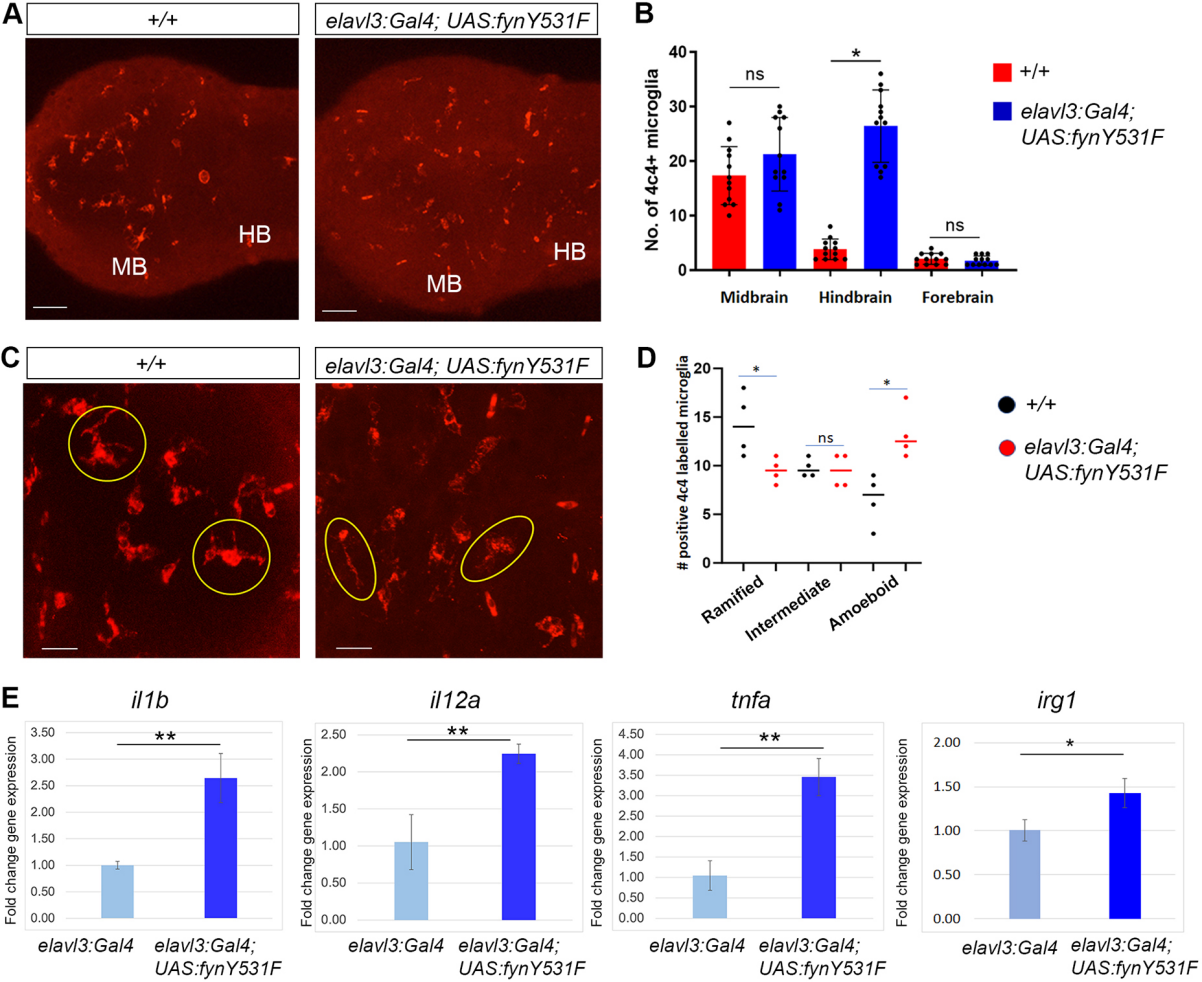
**Zebrafish neural FynY531F overexpression drives microglia activation and cytokine induction.** (A) 4C4 immunolabeling of microglia in wild-type +/+ and *elavl3:Gal4; UAS:fynY531F* forebrain and midbrain (MB)/hindbrain (HB). (B) Quantification of 4C4-labeled microglia in 5 dpf *+/+* and *elavl3:Gal4; UAS:fynY531F* larval brain (*n*=12). (C) Higher-magnification images of 4C4-labeled microglia in *+/+* and *elavl3:Gal4; UAS:fynY531F* larvae. Yellow circles outline microglia with ramified morphology; yellow ovals outline microglia with intermediate or amoeboid morphology. (D) Quantification of microglia with ramified, intermediate and amoeboid morphologies in 5 dpf *+/+* and *elavl3:Gal4; UAS:fynY531F* larval brain (*n*=4). (E) Reverse transcription quantitative PCR (RT-qPCR) of *il1b*, *il12a*, *tnfa* and *irg1* in RNA extracts from control *elavl3:Gal4* and *elavl3:Gal4; UAS:fynY531F* 5 dpf larvae (*n*=3 biological replicates for each genotype). Statistical analysis was performed with two-tailed unpaired Student’s *t*-test. Bars represent mean±s.e.m. ns, not significant; *P<0.05; **P<0.01. Scale bars: 50 µm (A); 20 µm (C).

To test whether FynY531F-driven dopaminergic neuron loss in the larval brain correlated with microglia activation, a marker of neuroinflammation, 5 dpf control +/+ and *elavl3:Gal4; UAS:fynY531Fis89* larvae were labeled with an anti-4C4 hybridoma supernatant (Fig. 3A). The 4C4 hybridoma supernatant recognizes galectin-3-binding protein and labels a subset of brain microglia (Becker and Becker, 2001; Chia et al., 2018; Mazzolini et al., 2020; Rovira et al., 2023). Compared to wild-type controls, *elavl3:Gal4; UAS:fynY531F* larvae did not show a significant difference in the number of 4C4-positive microglia in the forebrain (*P*=0.39) and midbrain (*P*=0.12), whereas the number detected in the hindbrain was elevated (*P*<0.0001) (Fig. 3A,B). Microglia show diverse morphologies that, in general, correlate with physiological state, ranging from ramified ‘resting’ to amoeboid ‘activated’, with numerous intermediate morphologies (Vidal-Itriago et al., 2022). The number of 4C4-positive microglia that showed ramified (Fig. 3C, yellow circles), intermediate and amoeboid (Fig. 3C, yellow ovals) morphologies was quantified in control and *elavl3:Gal4; UAS:fynY531F* larvae. In control, the number of ramified microglia was higher than the number of intermediate or amoeboid microglia (Fig. 3D). In contrast, the number of ramified microglia in *elavl3:Gal4; UAS:fynY531F* larval brains was slightly significantly lower (*P*=0.037), the number of intermediate microglia was not significantly different (*P*=0.809), and the number of amoeboid microglia was significantly higher (*P*=0.011) than those in control larval brain (Fig. 3D). The increase in the number of amoeboid microglia in *elavl3:Gal4; UAS:fynY531F* larval brains suggests that microglia become activated in response to elevated Fyn signaling.

To determine whether activated Fyn signaling led to induction of expression of genes indicative of inflammatory signaling and microglia activation, reverse transcription quantitative PCR (RT-qPCR) was used to examine the expression levels of the cytokine genes *il1b*, *il12a* and *tnfa* in dissected head tissue of 5 dpf control *elavl3:Gal4* and *elavl3:Gal4; UAS:fynY531F* larvae. *elavl3:Gal4; UAS:fyn531F* larvae showed a significant increase in the levels of *il1b* (*P*=0.003), *il12a* (*P*=0.006) and *tnfa* (*P*<0.002) (Fig. 3E). The gene encoding the activated microglia/inflammatory macrophage marker Aconitate decarboxylase 1/Immuno-responsive gene 1 (*acod1/irg1*) also showed significant elevation (*P*<0.02) in *elavl3:Gal4; UAS:fynY531F* larvae (Fig. 3E). Together, these results suggest that elevated neural Fyn signaling driving dopaminergic neuron loss correlates with induction of inflammatory cytokine expression and microglia activation.

### *elavl3:Gal4; UAS:fynY531F* dopaminergic neuron loss and microglia activation are dependent on Fyn kinase activity

**Fig. 4.**
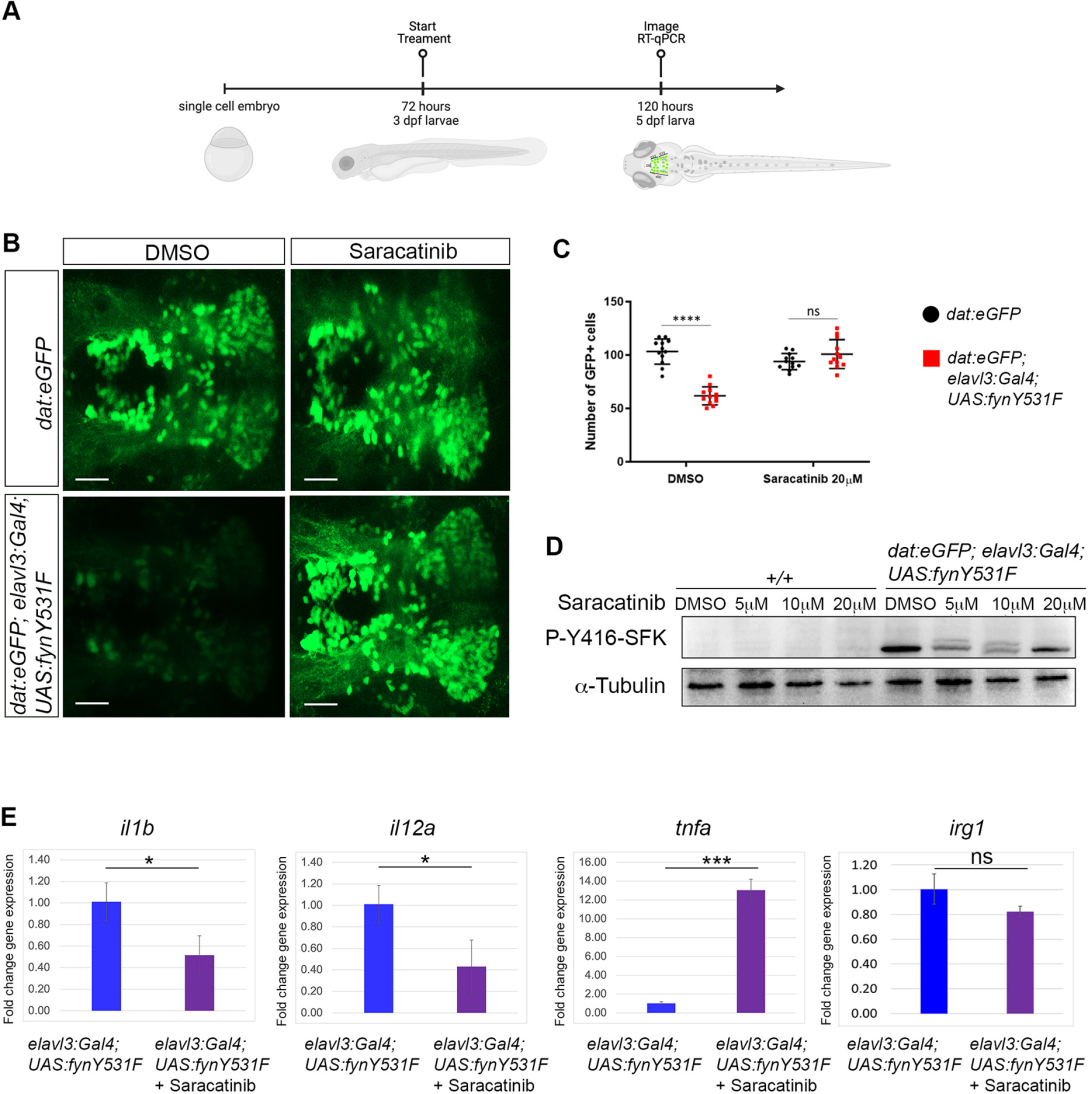
**Src family kinase inhibitor Saracatinib suppresses FynY531F-driven dopaminergic neuron loss, Fyn activation, and *il1b* and *il12a* cytokine induction.** (A) Time course of Saracatinib treatment beginning at 72 h post fertilization. (B) Live confocal imaging of vDC cluster eGFP dopaminergic neurons in 5 dpf control *dat:eGFP* and *elavl3:Gal4; UAS:fynY531F* larvae mock treated with DMSO or treated with 20 mM Saracatinib. (C) Quantification of vDC eGFP-positive neuronal cell bodies in 5 dpf control *dat:eGFP* and *dat:eGFP; elavl3:Gal4; UAS:fynY531F* larvae mock treated with DMSO or treated with 20 mM Saracatinib (*n*=12). Analysis was performed using two-way ANOVA with Tukey's multiple comparison. (D) Western blot of extracts from 5 dpf wild-type *+/+* and *dat:eGFP; elavl3:Gal4; UAS:fynY531F* larvae treated with DMSO and increasing amounts of Saracatinib, and probed with anti-Src family kinase P-Y416-SFK. Anti-acetylated tubulin and anti-alpha-tubulin were used as loading controls. (E) RT-qPCR of *il1b*, *il12a*, *tnfa* and *irg1* in RNA extracts from untreated and 20 µM Saracatinib-treated 5 dpf *elavl3:Gal4; UAS:fynY531F* larvae (*n*=3 biological replicates for all genotypes and conditions). This experiment was performed alongside the CAPE and Ac-YVAD-cmk experiments in Fig. 7C,D, using shared untreated controls. Control data in this panel are also shown in Fig. 7C,D. Statistical analysis was performed with two-tailed unpaired Student's *t*-test. Bars represent mean±s.e.m. ns, not significant; *P<0.05; ***P<0.001; ****P<0.0001. Scale bars: 50 µm.

In order to demonstrate that dopaminergic neuron loss, cytokine elevation and microglia activation are dependent on constitutive Fyn kinase signaling in the *elavl3:Gal4; UAS:fynY531F* model, we tested whether the Src family kinase inhibitor Saracatinib could suppress these cellular and molecular phenotypes (Fig. 4). We treated 3 dpf *dat:eGFP* control and *dat:eGFP; elavl3:Gal4; UAS:fynY531F* larvae continuously with 20 µM Saracatinib for 2 days and collected them at 5 dpf for live confocal imaging of vDC dopaminergic neurons and RNA extraction for RT-qPCR (Fig. 4A). Live imaging of eGFP expression in 5 dpf Saracatinib-treated larvae showed suppression of vDC neuron loss (Fig. 4B) and retention of vDC neuron numbers at levels equal to those in the control mock-treated (*P*=0.15) or Saracatinib-treated *dat:eGFP* (*P*=0.94) larvae (Fig. 4C). Western blot analysis of 5 dpf Saracatinib-treated larvae showed a reduction in the level of P-Y416-SFK in comparison to that in dimethyl sulfoxide (DMSO) mock-treated controls (Fig. 4D), indicating that FynY531F kinase activity was suppressed. RT-qPCR revealed that Saracatinib treatment led to a reduction in *il1b* and *il12a* expression in *elavl3:Gal4; UAS:fynY531F* larvae, demonstrating that elevated inflammatory cytokine levels were dependent on activated FynY531F signaling (Fig. 4E). In contrast, neither elevated *tnfa* nor *irg1* levels were suppressed by Saracatinib treatment of *elavl3:Gal4; UAS:fynY531F* larvae (Fig. 4E), possibly due to the non-specific action of the general Src family kinase inhibitor.

### RNA sequencing identifies Fyn-driven activation of Stat3, metabolic, oxidative stress and inflammatory signaling pathways

**Fig. 5.**
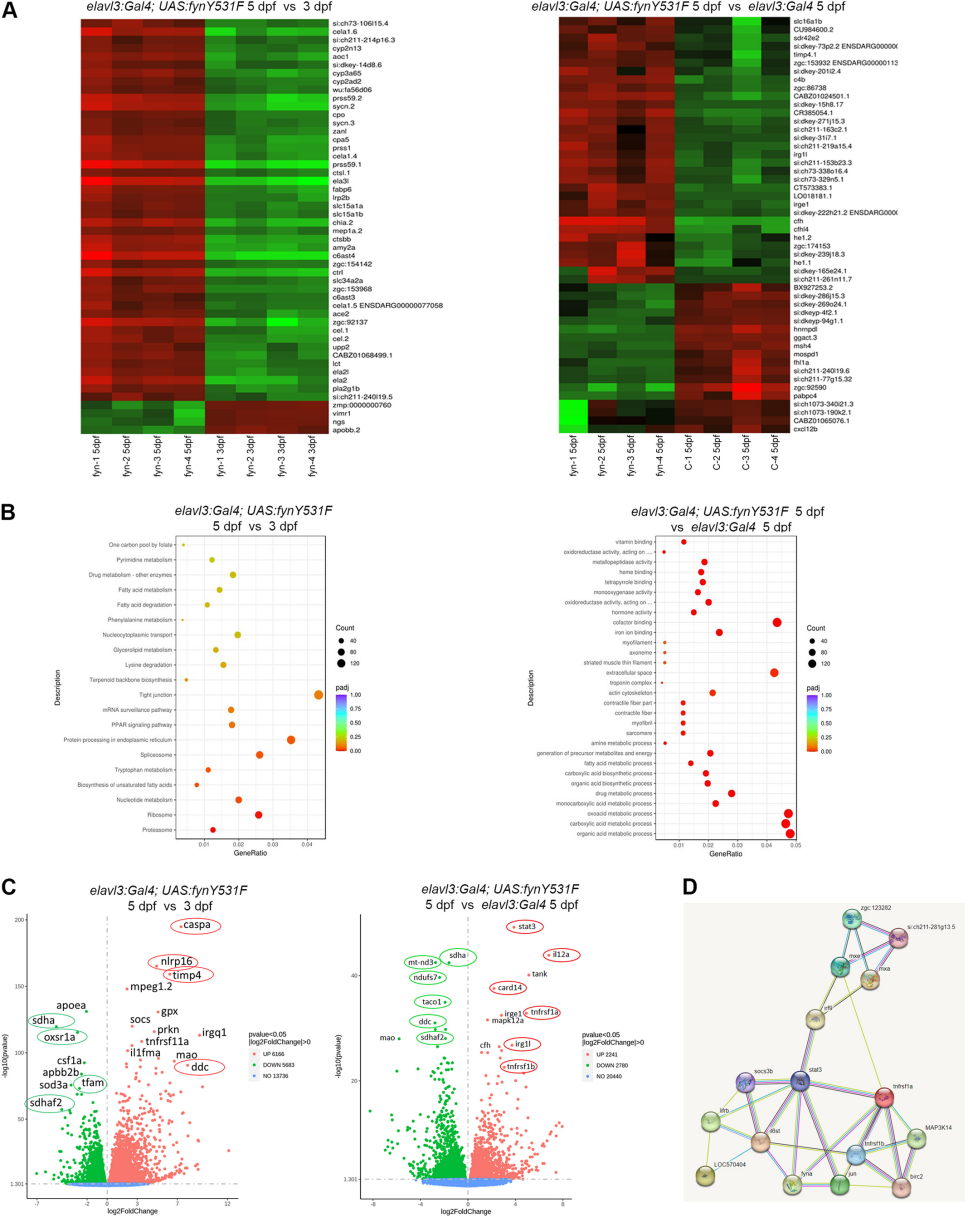
**Transcriptome analysis reveals that FynY531F signaling alters neuroprotective, oxidative and metabolic pathways, and identifies Stat3 as a downstream Fyn effector.** (A) Heatmaps of highest gene expression changes comparing bulk RNA sequencing from 5 dpf versus 3 dpf *elavl3:Gal4; UAS:fynY531F* larvae (*n*=4; left), and 5 dpf *elavl3:Gal4; UAS:fynY531F* versus 5 dpf *elavl3:Gal4* larvae (*n*=4; right). (B) Pathway analysis reveals altered metabolic pathways in 5 dpf versus 3 dpf *elavl3:Gal4; UAS: fynY531F* larvae (left) and 5 dpf *elavl3:Gal4; UAS:fynY531F* versus 5 dpf *elavl3:Gal4* larvae (right). (C) Volcano plots showing upregulated and downregulated genes in 5 dpf versus 3 dpf *elavl3:Gal4; UAS:fynY531F* larvae (left) and 5 dpf *elavl3:Gal4; UAS:fynY531F* versus 5 dpf *elavl3:Gal4* larvae (right). Nuclear-encoded mitochondrial genes *sdha*, *sdhaf2* and *taco1* are reduced. Elevated genes include *stat3*, *caspa*, *tngrsf1a/b* and *irg1l*. (D) STRING network analysis identifies Fyn interactions with Tnf-α and Stat3 signaling pathways in *elavl3:Gal4; UAS:fynY531F* larval transcriptome.

To identify altered pathways and potential downstream effectors of Fyn signaling in neurodegeneration, we performed bulk RNA sequencing (RNA-Seq) on RNA extracted from 3 dpf and 5 dpf control *elavl3:Gal4* and *elavl3:Gal4; UAS:fynY531F* larvae (Fig. 5; *n*=4 for each condition and genotype). The top 50 genes with highest changes in gene expression in 5 dpf versus 3 dpf *elavl3:Gal4; UAS:fynY531F* larvae included serine proteases *prss1* and *prss59.1*, and cathepsins *ctsbb* and *ctsl* (also known as *ctsll*), which are involved in proteolysis and metabolism in cytokine-related neuroinflammation (Ha et al., 2022; Jiang et al., 2022). The top genes in 5 dpf *elavl3:Gal4; UAS:fynY531F* versus control *elavl3:Gal4* larvae included *irg1l* (ortholog of *acod1/irg1*) and the Stat3 target *timp4.1* (Fig. 5A). Kyoto Encyclopedia of Genes and Genomes (KEGG) pathway analysis (Fig. 5B) indicated changes in electrical transmission, protein translation, metabolism of carbon, tryptophan and fatty acids, and the PPAR peroxisome proliferator-activated receptor pathway, which mediates inactivation of NF-κB during the inflammatory response (Korbecki et al., 2019). Volcano plots (Fig. 5C) comparing 3 dpf with 5 dpf *elavl3:Gal4; UAS:fynY531F* and 5 dpf *elavl3:Gal4; UAS:fynY531F* with control show reduced expression of the neuroprotective genes *apoea*, *sod3a* and *oxsr1a*. Apolipoprotein E (*APOE*) is essential for maintaining cholesterol homeostasis and neuronal function, and is a primary risk factor and therapeutic target in AD (Williams et al., 2020). Superoxide dismutase 3 (*SOD3*) encodes a key antioxidant enzyme protecting cells from oxidative stress-induced damage, a hallmark of neurodegeneration (Olufunmilayo et al., 2023). Oxidative stress responsive 1 (*OXSR1*) is essential for cellular resistance to oxidative stress, and its downregulation has been shown to precede the onset of neurodegeneration (Volkert and Crowley, 2020).

Consistent with oxidative stress as a factor in driving neurodegeneration, there was also a reduction in genes required for mitochondrial function (*taco1*, *mt-nd3*, *ndufs7*, *mao*, *tfam*) (Oktay et al., 2020; Richman et al., 2016) and oxidative phosphorylation (*sdha*, *sdhaf2*) (Fullerton et al., 2020). Elevated metabolic pathways identified by the KEGG analysis above suggested a compensatory mechanism for an energy deficit resulting from reduced mitochondrial function. Decreased dopa decarboxylase (*DDC*) suggests a disruption in dopaminergic neurotransmitter metabolism (Ramesh and Arachchige, 2023), which correlates with the loss of dopaminergic neurons in *elavl3:Gal4; UAS:fynY531F* larvae. Significant elevation of genes related to apoptosis or programmed cell death was not detected, although there was a reduction in Caspase-3 and apoptosis-related cysteine peptidase in 5 dpf versus 3 dpf *elavl3:Gal4; UAS:fynY531F* larvae. Together, these results suggest that Fyn signaling contributes to neurodegeneration through disruption of neuroprotective mechanisms and energy production.

Upregulated pathways consistent with the cytokine RT-qPCR gene expression analyses described above included elevated expression of components of inflammatory signaling (*nlrp16*, *caspa*, *card14*) and microglia activation (*irg1l*) (Fig. 5C). A significant increase was also detected in components of the Signal transducer and transcription activator (Stat) pathway, including *stat3*, *timp2b*, *timp4.1*, *socs3* (Fig. 5C) and Tnf-α receptors *tnfrsf1a/b*, the human homologs of which have been shown to be a direct transcriptional target of STAT3 in breast cancer cells (Egusquiaguirre et al., 2018). To reveal potential protein–protein interactions, STRING network analysis was performed, which identified connections between Fyn and Stat3 signaling (Fig. 5D), providing additional evidence for Stat3 as a potential novel downstream effector of Fyn. Together, these gene expression analyses indicate that Fyn-driven neurodegeneration may be due to defective neuroprotective mechanisms that correlate with oxidative stress, inflammatory cytokine production and Stat3 pathway activation.

### Stat3 is a novel Fyn downstream effector driving dopaminergic neuron loss and cytokine induction

**Fig. 6.**
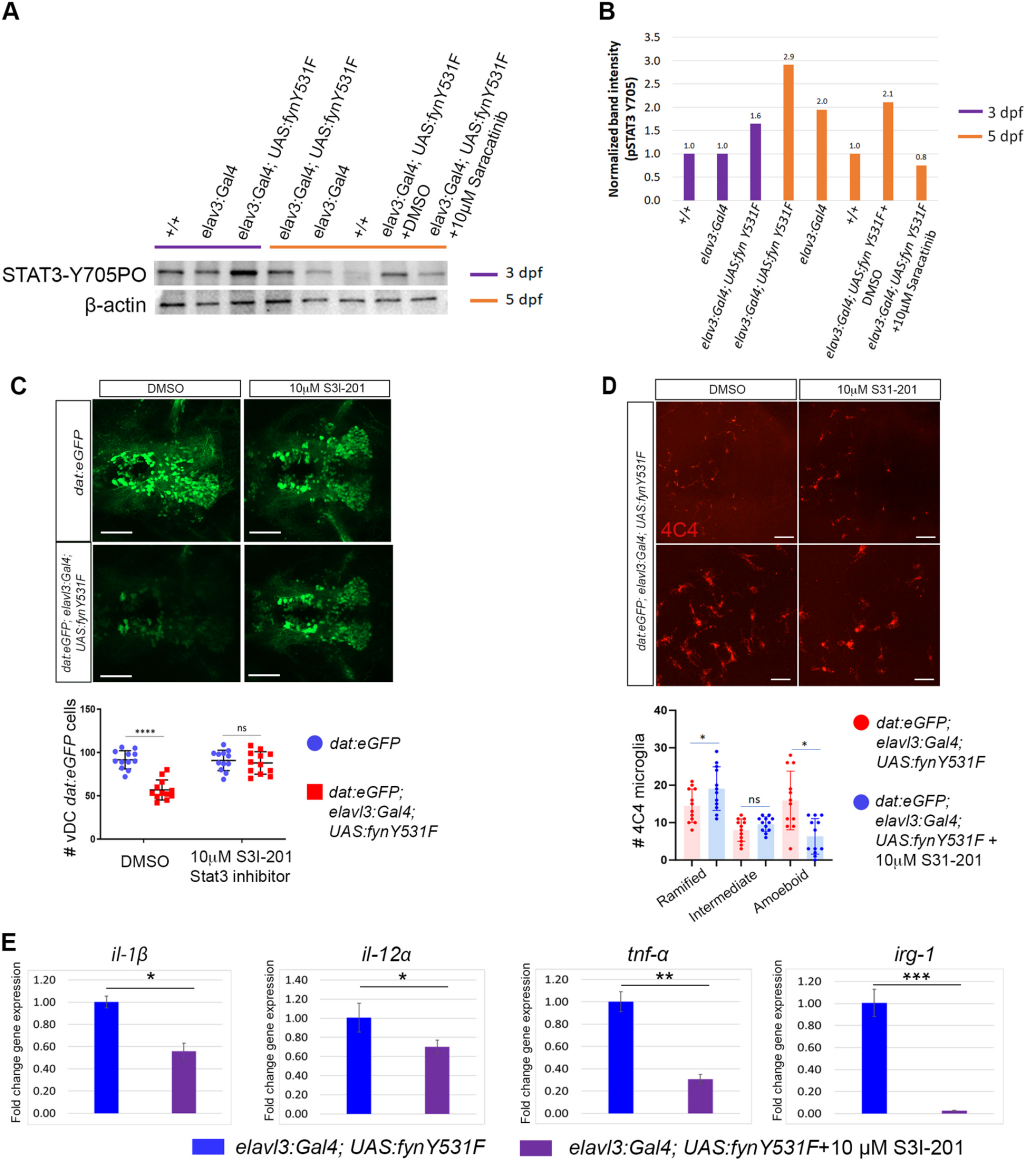
**Stat3 inhibition suppresses FynY531F-driven dopaminergic neuron degeneration and induction of *il1b*, *il12a*, *tnfa* and *irg1* expression.** (A) Western blot of 3 dpf and 5 dpf +/+, control *elavl3:Gal4* and *elavl3:Gal4; UAS:fynY531F* larval extracts probed with anti-Stat3-Y705-PO antibody shows increased Stat3-Y705 phosphorylation in *elavl3:Gal4; UAS:fynY531F* larvae. *elavl3:Gal4; UAS:fynY531F* larvae treated from 3 dpf to 5 dpf with 10 µM Saracatinib (Src family inhibitor) show reduced Stat3-Y705 phosphorylation. Blot was probed with anti-Gapdh and anti-β-actin as protein-loading controls. (B) Quantification of band intensities on Stat3-Y750-PO western blot. (C) Top: live confocal imaging of vDC eGFP dopaminergic neurons in 5 dpf control *dat:eGFP* and *dat:eGFP;elav:Gal4; UAS:fynY531F* larvae show rescue of dopaminergic neuron loss after treatment with 10 µM S3I-201 (Stat3 inhibitor). Bottom: quantification of vDC eGFP dopaminergic neuron number in 5 dpf control *dat:eGFP* and *dat:eGFP;elav:Gal4; UAS:fynY531F* larvae after treatment with 10 µM S3I-201 (*n*=12). Statistical analysis was performed using two-way ANOVA with Tukey's multiple comparison. (D) Top: confocal imaging of fixed, whole-mount anti-4C4 immunolabeling in brain of 5 dpf control *dat:eGFP* and *dat:eGFP;elav:Gal4; UAS:fynY531F* larvae at low magnification (top row) and high magnification (bottom row) after treatment with vehicle (DMSO) and Stat3 inhibitor (S3I-201). Bottom: quantification of 4C4-positive microglia with ramified, intermediate or amoeboid morphology number in 5 dpf control *dat:eGFP* and *dat:eGFP;elav:Gal4; UAS:fynY531F* larvae and after treatment with 10 mM Stat3 inhibitor S3I-201 (*n*=12). Analysis was performed using two-way ANOVA with Tukey's multiple comparison. (E) RT-qPCR of *il1b*, *il12a*, *tnfa*, and *irg1* in RNA extracts from untreated and 10 µM S3I-201-treated 5 dpf *elavl3:Gal4; UAS:fynY531F* larvae (*n*=3 biological replicates for each genotype). Statistical analysis was performed with two-tailed unpaired Student's *t*-test. Bars represent mean±s.e.m. ns, not significant; *P<0.05; **P<0.01; ***P<0.001; ****P<0.0001. Scale bars: 100 µm (C); 20 µm (D).

Differential gene expression in the FynY531F *elavl3:Gal4; UAS:fynY531F* model identified Stat3 as a potential downstream pathway activated by Fyn signaling. In cultured human melanoma cells, FYN kinase has been shown to phosphorylate STAT3 on Tyr705 (Tang et al., 2020). To test whether Fyn signaling drove Stat3 activation in the zebrafish Fyny531F model, western blotting of wild-type +/+, control *elavl3:Gal4* and *elavl3:Gal4; UAS:fynY531F* larvae was performed with an anti-Stat-Y705-PO antibody (Fig. 6A). At 3 dpf and 5 dpf, elevated levels of Stat-Y705-PO were detected in *elavl3:Gal4; UAS:fynY531F* larvae compared to those in wild-type and control larvae (Fig. 6A,B). Treatment of *elavl3:Gal4; UAS:fynY531F* from 3 dpf to 5 dpf with 10 µM Saracatinib (Src family inhibitor) inhibited Stat-Y705-PO levels, which remained similar to those detected in control *elavl3:Gal4* larvae (Fig. 6A,B). These results showed that FynY31F signaling drives Stat3-Y705 phosphorylation, which is dependent on Fyn kinase activity.

To determine whether dopaminergic neuron loss in the FynY531F model was dependent on Stat3 activation, *dat:eGFP; elavl3:Gal4; UAS:fynY531F* larvae were treated with 10 µM S3I-201 (Stat3 inhibitor), followed by quantification of the vDC *dat:eGFP* cluster (Fig. 6C). S3I-201 treatment from 3 dpf to 5 dpf suppressed *dat:eGFP* neuron loss in *elavl3:Gal4; UAS:fynY531F* larvae (Fig. 6C). The number of *dat:eGFP*-positive cells in treated *elavl3:Gal4; UAS:fynY531F* larvae was not significantly different from that in untreated control (*P*=0.99) or treated control (*P*=0.92) larvae (Fig. 6C). Together with the results shown above, this analysis indicates that Stat3 is a downstream effector of Fyn signaling, which mediates dopaminergic neuron loss.

We next examined whether microglia activation and elevated cytokine levels in *elavl3:Gal4; UAS:fynY531F* larvae were dependent on Stat3 activation. *dat:eGFP; elavl3:Gal4; UAS:fynY531F* larvae were treated with 10 µM S3I-201, fixed and labeled with anti-4C4 antibody (Fig. 6D). The number of microglia with ramified, intermediate and amoeboid morphologies were quantified in untreated control *dat:eGFP; elavl3:Gal4; UAS:fynY531F* and treated *dat:eGFP; elavl3:Gal4; UAS:fynY531F* larvae (Fig. 6D). In larvae treated with S3I-201, the number of ramified microglia showed a slightly significant increase (*P*=0.042), there was no significant change in the number of intermediate microglia (*P*=0.140), and the number of amoeboid microglia showed a significant decrease, relative to that in control (*P*=0.0015) (Fig. 6D). RT-qPCR of *elavl3:Gal4; UAS:fynY531F* larvae treated with 10 µM S3I-201 showed that treatment with the Stat3 inhibitor suppressed elevated levels of cytokines *il1b* (*P*<0.05), *il12a* (*P*<0.04) and *tnfa* (*P*<0.001), and microglia activation marker *irg1* (*P*>0.0001) (Fig. 6E). Together, these results are consistent with Fyn signaling driving the Stat3 activation and induction of inflammatory cytokine expression that correlates with dopaminergic neuron loss and microglia activation.

### FynY531F-induced dopaminergic neuron loss and cytokine elevation depend on NF-κB inflammatory signaling

**Fig. 7.**
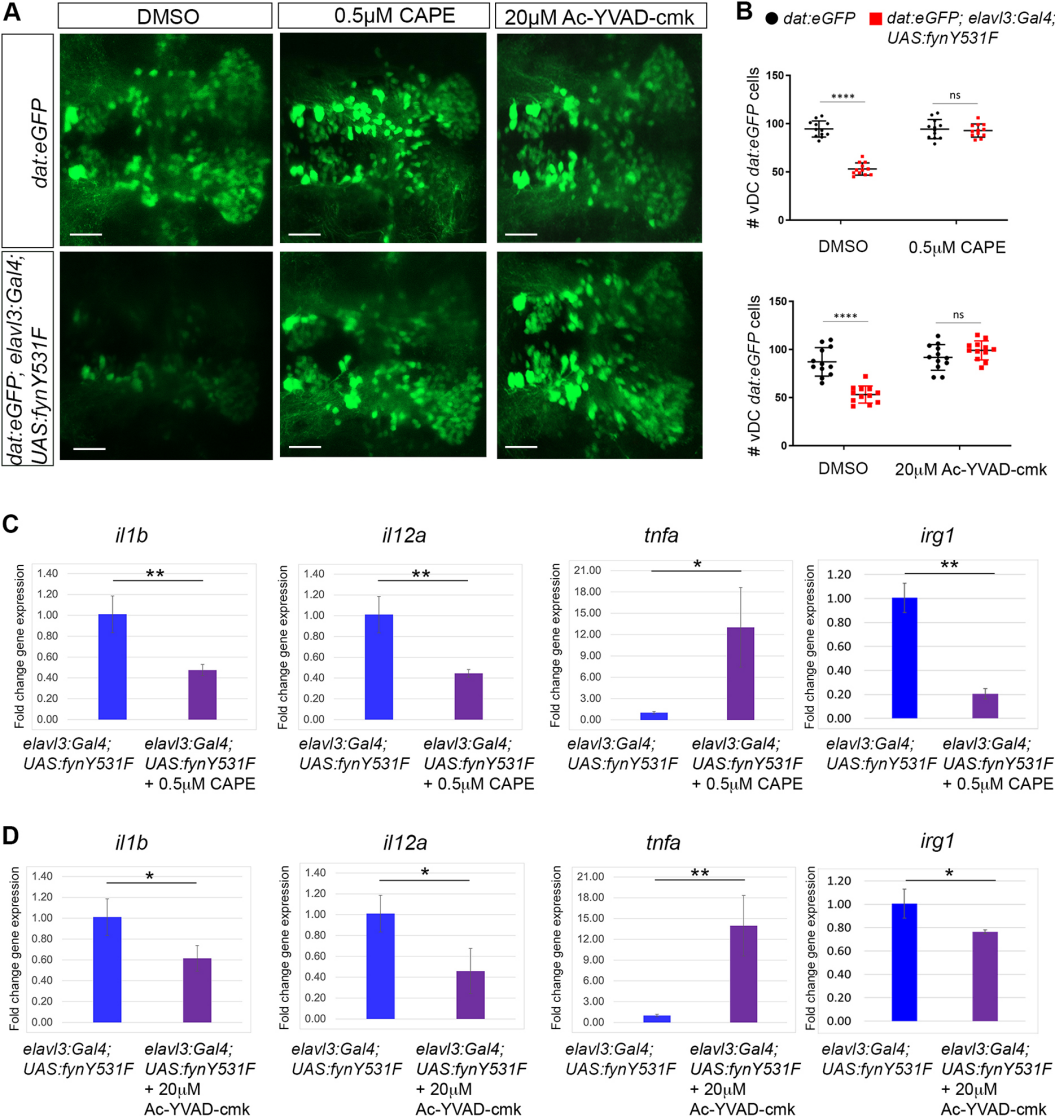
**NF-κB and Caspase-1 inhibition suppresses FynY531F-driven dopaminergic neuron loss and induction of *il1b*, *il12a* and *irg1* expression.** (A) Live confocal imaging of vDC eGFP-positive dopaminergic neurons in 5 dpf control *dat:eGFP* and *dat:eGFP;elav:Gal4; UAS:fynY531F* larvae show rescue of dopaminergic neuron loss after treatment with 0.5 µM caffeic acid phenethyl ester (CAPE; NF-κB inhibitor) and 20 µM Ac-YVAD-cmk (Caspase-1 inhibitor). (B) Quantification of vDC eGFP-positive cells (*n*=12). Analysis was performed using two-way ANOVA with Tukey's multiple comparison. (C,D) RT-qPCR revealed significantly reduced levels of *il1b*, *il12a* and *irg1* after treatment of *dat:eGFP;elav:Gal4; UAS:fynY531F* larvae with 0.5 µM CAPE (C) and 20 µM Ac-YVAD-cmk (D). *tnfa* was increased after treatment with 0.5 µM CAPE and 20 µM Ac-YVAD-cmk (*n*=3 biological replicates for each genotype and condition). These experiments were performed together with the Saracatinib experiment in Fig. 4E, using shared untreated controls. Control data in these panels are also shown in Fig. 4E. Statistical analysis was performed with two-tailed unpaired Student's *t*-test. Bars represent mean±s.e.m. ns, not significant; *P<0.05; **P<0.01; ****P<0.0001. Scale bars: 50 µm.

The results presented above indicate that Fyn signaling drives induction of inflammatory cytokines *il1b* and *il12a*, which are known to be expressed by activated microglia via the NF-κB pathway (Wang et al., 2015). To test whether FynY531F-driven dopaminergic neuron loss was dependent on the NF-κB inflammatory signaling pathway, *dat:eGFP*; *elavl3:Gal4; UAS:fynY531F* larvae were treated at 3 dpf with NF-κB and Caspase-1 inhibitors, and collected at 5 dpf for live imaging and quantification of vDC dopaminergic neurons. Treatment of *dat:eGFP*; *elavl3:Gal4; UAS:fynY531F* larvae with 0.5 µM caffeic acid phenethyl ester (CAPE; NF-κB inhibitor) suppressed vDC dopaminergic neuron loss (Fig. 7A). The numbers of eGFP-positive vDC neurons in treated larvae showed no significant difference from those in control *dat:eGFP* larvae (*P*=0.96) (Fig. 7B). Treatment of *dat:eGFP*; *elavl3:Gal4; UAS:fynY531F* larvae with 20 µM Ac-YVAD-cmk, a Caspase-1 inhibitor, also suppressed vDC dopaminergic neuron loss compared to that in control larvae (Fig. 7A,B, *P*=0.08). These results indicate that constitutive FynY531F kinase signaling driving dopaminergic neuron loss is mediated by activation of the inflammatory signaling pathway NF-κB/Caspase-1.

RT-qPCR was used to determine whether *elavl3:Gal4; UAS:fynY531F*-elevated inflammatory cytokine expression was dependent on the NF-κB/Caspase-1 pathway. Treatment of *elavl3:Gal4; UAS:fynY531F* larvae with 0.5 µM CAPE suppressed the increase in *il1b*, *il12a* and *irg1* expression (Fig. 7C). The results with 20 µM Ac-YVAD-cmk were similar, with a slightly less significant difference in expression level compared to that in untreated *elavl3:Gal4; UAS:fynY531F* larvae (Fig. 7D). Neither CAPE nor Ac-YVAD-cmk suppressed the elevation in *tnfa* expression in *elavl3:Gal4; UAS:fynY531F* larvae. Together, these results suggest that microglia and NF-κB/Caspase-1 pathway activation occur in response to an external inflammatory signal, which may originate in degenerating dopaminergic neurons.

### Stat3 and NF-κB synergize in Fyn-driven dopaminergic neuron loss

**Fig. 8.**
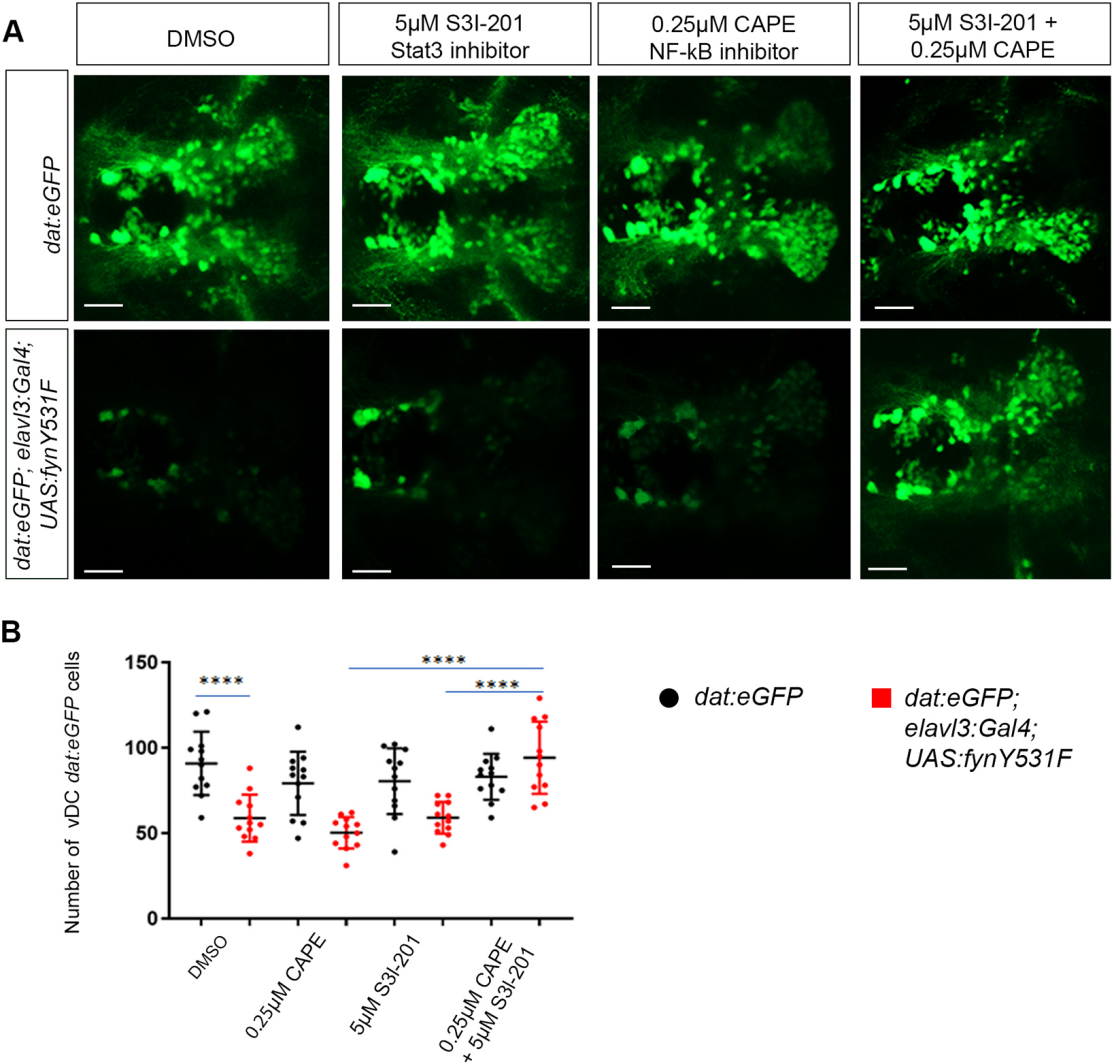
**Dual inhibition of NF-κB and Stat3 acts synergistically to suppress FynY531F-driven dopaminergic neuron degeneration.** (A) Live confocal imaging of vDC eGFP dopaminergic neurons in 5 dpf control *dat:eGFP* and *dat:eGFP;elav:Gal4; UAS:fynY531F* larvae after treatment with mock DMSO, 5 µM S3I-201, 0.52 µM CAPE, and 5 µM S3I-201 plus 0.25 µM CAPE. (B) Quantification of vDC eGFP dopaminergic neuron number in 5 dpf control *dat:eGFP* and *dat:eGFP;elav:Gal4; UAS:fynY531F* larvae after treatment with mock DMSO, 5 µM S3I-201, 0.25 µM CAPE, and 5 µM S3I-201 plus 0.25 µM CAPE (*n*=12). Analysis was performed using two-way ANOVA with Tukey's multiple comparison. Bars represent mean±s.e.m. ****P<0.0001. Scale bars: 50 µm.

To examine the interaction of Stat3 and NF-κB pathways in Fyn-driven dopaminergic neuron loss, *dat:eGFP; elavl3:Gal4; UAS:fynY531F* larvae were treated either alone or with a combination of Stat3 and NF-κB inhibitors (Fig. 8A). Dual treatment of larvae with 10 µM S31-301 and 0.5 µM CAPE was highly toxic and resulted in lethality at 5 dpf. Therefore, the amount of each inhibitor was reduced by half to test for interaction of the two pathways. Control *dat:*eGFP and *dat:eGFP; elavl3:Gal4; UAS:fynY531F* larvae were treated with vehicle DMSO, 5 µM S31-301, 0.25 µM CAPE, or 5 µM S31-301 plus 0.25 µM CAPE. Treatment of *dat:eGFP; elavl3:Gal4; UAS:fynY531F* larvae with either inhibitor alone did not significantly suppress the loss of *dat:eGFP*-labeled vDC neurons compared to that in DMSO-treated *dat:eGFP; elavl3:Gal4; UAS:fynY531F* larvae (5 µM S31-301, *P*=0.9726; 0.25 µM CAPE, *P*=0.0861) (Fig. 8A,B). Dual treatment with 5 µM S31-301 and 0.25 µM CAPE suppressed the loss of *dat:eGFP* signal to a significantly higher level than either inhibitor alone (Fig. 8A,B) (5 µM S31-301 versus 5 µM S31-301 plus 0.25 µM CAPE, *P*<0.0001; 0.5 µM CAPE versus 5 µM S31-301 plus 0.25 µM CAPE, *P*<0.0001). These results indicate that Stat3 and NF-κB pathways act synergistically to drive dopaminergic neurodegeneration in response to Fyn signaling.

## DISCUSSION

Here, we describe a novel zebrafish model of activated Fyn signaling that demonstrates a neural-specific role for Fyn-driven neurodegeneration. The zebrafish Fyn model provides *in vivo* evidence consistent with a role for FYN signaling in the pathology of neurodegenerative disorders and reports of elevated FYN signaling in patient brain tissue (Guglietti et al., 2024; Low et al., 2021; Panicker et al., 2019). In the zebrafish Fyn model, activated Fyn drives inflammatory cytokine production, leading to dopaminergic neuron loss, mitochondrial accumulation and microglia activation. Chemical inhibition demonstrated that Fyn drives neurodegeneration through activation of Stat3 and NF-κB/Caspase 1, which synergize in dopaminergic neuron loss. Although both Stat3 and NF-κB were required for induction of *il1b* and *il12a*, *tnfa* elevation was only dependent on Stat3 activation. Our study suggests a model in which Fyn drives production of *tnfa* through activation of Stat3 signaling in neurons. Release of *tnfa* may contribute to microglia activation, driving production of inflammatory cytokines *il1b* and *il12a*, which in turn stimulate a sustained neuroinflammatory response correlated with mitochondrial accumulation and neuron loss (Fig. S1).

Activated Fyn signaling led to dopaminergic neuron loss over the course of 3 dpf to 5 dpf, after the onset of neural differentiation in the larval brain. Given the critical role of mitochondrial dysfunction in neurodegenerative disease (Klemmensen et al., 2024), we examined whether neuron loss correlated with an observable change in mitochondria. We used the dopaminergic neuron mitochondrial fluorescence reporter *dat:mitoRFP*, which shows a reduction in signal in larvae treated with the neurotoxin MPTP (Noble et al., 2015). Neural function is dependent on the active transport of mitochondria along axonal microtubules to both the cell body (Mandal et al., 2021) and the energy-intensive neural synapse (Vos et al., 2010). The increase in *dat:mitoRFP* signal in dopaminergic cell bodies in 3 dpf larvae, before the loss of *dat:eGFP*-labeled dopaminergic neurons, suggested that Fyn activation disrupts neuron health and organelle transport in the axon. Changes in gene expression indicated that there may also be defects in mitochondrial respiratory complex function (*sdha*, *sdhaf2*, *mt-nd3*, *ndufs7*, *taco1*) (Borna et al., 2024; Fullerton et al., 2020; Machado et al., 2016; Oktay et al., 2020; Richman et al., 2016) or biogenesis (*tfam*) (Kang et al., 2007; Stiles et al., 2016). Fyn-driven dopaminergic neuron loss did not correlate with an increase in programmed cell death pathway genes. These observations are distinct from reports of Src family/Fyn kinase phosphorylation of PKC-δ leading to rat dopaminergic neuron oxidative stress and cell death through caspase-mediated apoptosis (Kaul et al., 2005; Saminathan et al., 2011). It is possible that direct inhibition of mitochondrial protein translation by Fyn phosphorylation of mitochondrial elongation factor EF-Tu_mt_ (also known as TUFM) underlies defects in mitochondrial function (Koc et al., 2023). Overall, these analyses are consistent with Fyn activation impacting neuron health and survival through a mechanism distinct from apoptosis-induced programmed cell death. Additional studies to examine mitochondrial biogenesis, integrity and respiratory function are needed to experimentally determine whether mitochondrial dysfunction is a contributing factor in Fyn-driven neurodegeneration.

Transcriptome analysis of the zebrafish Fyn model identified Stat3 as a novel downstream effector of Fyn signaling in neurodegeneration. Like Fyn, Stat3 signaling has been implicated in neurodegeneration. FYN and STAT3 were identified as potential AD biomarkers in young APOE ε4 individuals (Roberts et al., 2021). *In vitro* analyses demonstrated that Stat3 and Fyn inhibitors suppressed AD-related phenotypes of lipopolysaccharide-induced neuroinflammation, tau phosphorylation and Aβ secretion (Roberts et al., 2021). STAT3-PO is observed in AD patient postmortem brain tissue and in mouse APP/PS1 AD model brain, and mediates *in vitro* antibody-induced neuron cell death (Wan et al., 2010). Astrogliosis in the mouse APP/PS1 AD model is dependent on Stat3 (Ben Haim et al., 2015; Reichenbach et al., 2019). STAT3-Y705 phosphorylation has been shown to be regulated by SRC kinases in cultured tumor cells (Garcia et al., 2001; Ram and Iyengar, 2001; Silva, 2004), and Fyn is required for Stat3-PO activation in T-cell receptor signaling and T-cell differentiation (Qin et al., 2024). Our model suggests that, *in vivo*, Fyn and Stat3 function in the same pathway to drive neuroinflammation and neurodegeneration, with Stat3 activation dependent on Fyn signaling.

Dual chemical inhibition revealed that Stat3 and NF-κB pathways synergize in dopaminergic neuron loss in the Fyn model. Chemical inhibition of Stat3 significantly suppressed all three elevated cytokines – *il1b*, *il12a* and *tnfa* – supporting a role for Stat3 in induction of multiple inflammatory signaling pathways and microglia activation. Although inhibiting NF-κB and Caspase-1 suppressed expression of *il1b* and *il12a*, it did not suppress *tnfa* induction, suggesting a temporal and spatial separation of activation of the Stat3 and NF-κB pathways. However, Stat3 gene targets elevated in the Fyn neurodegeneration model included *tnfrsf1a*; its human homolog (*TNFRSF1A*) encodes a receptor for TNF-α (also known as TNF) and is associated with activation of the NF-κB pathway in breast cancer (Egusquiaguirre et al., 2018). STAT3 and NF-κB pathways have also previously been shown to interact in hepatic cells. STAT3 and RELA/p65 formed a complex in HepG2 hepatoblastoma cells stimulated with IL-1 and IL-6 (Hagihara et al., 2005), and NF-κB RELA/p65 homodimers were shown to cooperate with STAT3 in Hep3B cells in response to IL-1 (Yoshida et al., 2004). In cultured microglia, FYN activation was shown to lead to cytokine production through activation of PKC-δ/NF-κB/caspase-1 inflammasome signaling (Panicker et al., 2015, 2019). In our *in vivo* model, the possibility of FYN directly activating the NF-κB pathway in neurons cannot be excluded, nor can indirect activation of Stat3 in microglia leading to stimulation of the NF-κB pathway. Microglia are known to produce TNF-α when activated through the JAK/Stat pathway (Yin et al., 2018). Distinguishing these possibilities would require cell type-specific inhibition to determine whether Stat3 and NF-κB/Caspase-1 pathways are functioning in dopaminergic neurons, microglia or both. Nevertheless, our findings suggest an interplay between Fyn-driven cytokine production in neurons and activated microglia, potentially creating a feedback loop that fuels persistent neuroinflammation and neurodegeneration.

Our *in vivo* zebrafish model of neural Fyn signaling reveals that Fyn drives dopaminergic loss through inflammatory cytokine production and microglia activation, and unveils Stat3 as a potential novel downstream Fyn effector. The synergistic effect of Stat3 and NF-κB inhibition in suppressing Fyn-driven dopaminergic loss support the contribution of both pathways in mediating Fyn neurodegeneration. Further investigation is necessary to identify the role of Stat3 and its cellular targets in driving dopaminergic neuron loss and mitochondrial dysfunction in response to Fyn signaling.

## MATERIALS AND METHODS

### Ethics declarations and approval for animal experiments

Use of zebrafish for research in this study was performed according to the Guidelines for Ethical Conduct in the Care and Use of Animals (American Psychological Association, 1986), and carried out in accordance with Iowa State University Animal Care and Use Committee-approved protocols (IACUC-21-281 and IBC-21-117). All methods involving zebrafish were in compliance with the American Veterinary Medical Association (Percie du Sert et al., 2020), Animal Research: Reporting of *In Vivo* Experiments (Percie du Sert et al., 2020) and National Institutes of Health [National Research Council (US) Committee for the Update of the Guide for the Care and Use of Laboratory Animals, 2011] guidelines for the humane use of animals in research.

### Zebrafish maintenance and transgenic strains

Zebrafish *Danio rerio* used in this study were housed in the Roy J. Carver Charitable Trust Zebrafish Facility at Iowa State University. Fish were maintained at 28.5°C on an Aquaneering aquaculture system with a 14 h light/10 h dark-light/dark cycle. Embryos were collected from natural spawning and were raised in E3 embryo medium (13 mM NaCl, 0.5 mM KCl, 0.02 mM Na_2_HPO_4_, 0.04 mM KH_2_PO_4_, 1.3 mM CaCl_2_, 1.0 mM MgSO_4_, 4.2 mM NaHCO_3_, pH 7.0). Wild-type WIK zebrafish were obtained from the Zebrafish International Resource Center. Transgenic zebrafish previously described and used in this study include the dopaminergic neuron eGFP and mito-RFP reporters *Tg*(*dat:eGFP)* (*dat:eGFP*) (Xi et al., 2011b) and *Tg(dat:tom20 MLS-mCherry)* (*dat:mitoRFP*) (Noble et al., 2015), and the pan-neuronal Gal4 driver *Tg(elavl3:Gal4-VP16)nns6* (Kimura et al., 2008).

### Isolation of *Tg(UAS:fynY531F)* transgenic lines

The 1614 bp *fyna* (ENSDARG00000011370) wild-type and Y531F mutant cDNAs were amplified by RT-qPCR from RNA isolated from 3 dpf embryos using the primers listed in Table S1. The forward and reverse primers incorporated KpnI and BssHII restriction enzyme sites, respectively. Five-hundred nanograms of RNA was as a template for reverse transcription with Superscript II (Invitrogen, 11752), followed by amplification with KOD polymerase (Sigma-Aldrich, 71842). Mutant *fynY531F* was directionally cloned into the transposon vector *Tol2(14XUAS, gcry1:eGFP)* (Balciuniene et al., 2013) to build the *Tol2 (UAS:fynY531F; gcry1:eGFP)* construct. *Tol2* transposase mRNA was *in vitro* transcribed from 1 μg linear *pT3TS-Tol2* plasmid (Balciunas et al., 2006) to generate capped, polyadenylated mRNA using a T3 mMessage mMachine High Yield Capped RNA Transcription Kit (Thermo Fisher Scientific, AM 1348). *Tol2* mRNA was purified using an RNA Clean & Concentrator-5 Kit (ZYMO, R1015) and eluted in RNase-free water. *Tg(Tol2<UAS:fynY531F>)is89* and *Tg(Tol2<UAS:fynY531F>)is90* lines were isolated by co-injection of 50 pg *Tol2* mRNA and 25 pg transposon vector into one-cell WIK embryos. Five *Tg(Tol2<UAS:fynY531F>)* F0 adults were screened to identify two independent founders transmitting *Tol2* integration through the germline. Individual F1 adults were used to generate F2 fish, and a single F2 adult showing Mendelian segregation of transmitted alleles was outcrossed to WIK to establish the independent lines *Tg(Tol2(14XUAS:fynaY531F, gcry1:eGFP>)is89* and *Tg(Tol2(14XUAS:fynaY531F, gcry1:eGFP>)is90*.

### Behavioral analysis

Zebrafish larvae were monitored for swimming behavior using a ZebraBox monitoring system and ZebraLab software (ViewPoint Behavior Technology). Larvae at 4 dpf were placed in a 48-well plate in E3 embryo medium. At 5 dpf, larvae were placed in the viewing chamber and acclimated for 30 min before recording. Locomotion was recorded for 5 h under alternating light/dark conditions in 15 min intervals. Larval movement, velocity and distance were recorded each minute, and the data were analyzed and plotted with ZebraLab software.

### Chemical inhibitor treatment assays

For chemical inhibitor assays, ten larvae of each genotype at 3 dpf were placed in a well in a six-well plate in 3 ml embryo medium containing 0.003% 0.2 M phenylthiourea and the designated inhibitor diluted to the final concentration, or DMSO as a vehicle control. The larvae were placed in a 28.5°C incubator. The chemical inhibitor or DMSO solution was replaced with fresh solution in the morning of 4 dpf and 5 dpf. Caspase-1 inhibitor InvitroFit™ Ac-YVAD-cmk (Garcia-Calvo et al., 1998) (InvivoGen, inh-yvad) was dissolved in DMSO and used at 20 µM final working concentration. NF-κB inhibitor CAPE (Natarajan et al., 1996) (Apexbio, B1644) in DMSO was used at 0.5 µM final working concentration. Src family kinase inhibitor Saracatinib AZD0530 (Green et al., 2009) (MedChemExpress, HY-1-234) in DMSO was used at 20 µM final working concentration. Stat3-PO inhibitor S31-301 was used at a final concentration of 10 µM. For dual inhibitor assays, CAPE and S31-301 were used a final concentration of 0.25 µM and 5 µM, respectively.

### RNA isolation, RT-qPCR and RNA-Seq

RT-qPCR experiments were designed and carried out according to updated Minimum Information for Publication of Quantitative Real-Time PCR Experiments guidelines (Bustin et al., 2009; Taylor et al., 2019). For RT-qPCR, head tissue was dissected from 30 larvae at 5 dpf per biological replicate and placed in DNA/RNA Shield (Zymo Research, R1100-50) and homogenized using a disposable pestle. RNA extraction was performed according to the manufacturer's instructions. We used 500 ng total RNA as a template with a Luna Universal One-step RT-qPCR Kit (New England Biolabs, E3005L). For each condition, three biological replicates with two technical replicate RT-qPCR reactions were run on a CFX96 Connect Real-Time System (Bio-Rad). The number of *il1b*, *il12a*, *tnfa* and *irg1* transcripts was quantified using the comparative CT method (ΔΔCT) and normalized using *rps6kb1b* reference gene. The oligonucleotide primers used for RT-qPCR are listed in Table S1. For RNA-Seq, ten whole larvae were pooled for each biological replicate, and four biological replicates were used for each condition. Library construction, PE150 next-generation sequencing, and differential gene expression were performed at Novogene using DeSeq2. Differential gene expression data were analyzed with online software for heat map generation and KEGG analysis, and STRING analysis.

### Western blotting

Protein extracts were generated from zebrafish larvae placed in lysis buffer [50 mM Tris-HCl pH 7.5, 150 mM NaCl, 5 mM MgCl_2_, 1% Triton X-100, 0.5% NP-40 containing 1× Halt Protease Inhibitor single-use cocktail (Thermo Fisher Scientific, 78430)] and ground with a disposable pestle. SDS-PAGE of protein extracts was performed with a Bio-Rad Mini-Protean gel system using Mini-PROTEAN TGX Precast 4-15% polyacrylamide gels (Bio-Rad, 4561083) and blotted to an Immun-Blot LF PVDF membrane (Bio-Rad. 1620174). Blots were blocked with Blot-Qualified BSA (Sigma-Aldrich, A7906) and probed with the following primary antibodies: anti-416-PO Srk kinase family rabbit polyclonal (Cell Signaling Technology, 6943T) at 1:1000, anti-Stat3-Y705PO XP rabbit monoclonal antibody (Cell Signaling Technology, 9145) and anti-Gapdh mouse monoclonal antibody (Proteintech, 60004-1-Ig). Goat anti-mouse IgG horseradish peroxidase (Invitrogen, 31430) and goat anti-rabbit IgG horseradish peroxidase (Invitrogen, 31460) secondary antibodies were used at 1:10,000 dilution. Western blots were developed with SuperSignal West Dura Extended Duration Substrate (Thermo Fisher Scientific, 34075) and imaged on an iBright system (Thermo Fisher Scientific, FL1500).

### Zebrafish immunolocalization and live confocal imaging

Zebrafish larvae fixation, embedding, sectioning and immunolabeling were as described previously (Schultz et al., 2018). Embryos were incubated in 0.003% 1-phenyl 2-thiourea (Sigma-Aldrich, P7629) in E3 embryo medium to inhibit pigment synthesis. Larvae were euthanized in 160 µg/ml ethyl 3-aminobenzoate methanesulfonate and fixed in 4% paraformaldehyde overnight at 4°C or in 2% trichloroacetic acid for 3 h at room temperature. Whole-mount fixed larvae were labeled to visualize macrophages and microglia with the mouse hybridoma supernatant 4C4 (ECACC 92092321, A. Dowding, King's College London, London, UK; a gift from Dr Diana Mitchell, University of Idaho, Moscow, ID, USA) at 1:100 dilution. Rabbit polyclonal anti-acetylated alpha tubulin antibody (Invitrogen, PA5-105102) was used at 1:500. Secondary antibodies Alexa Flour 594 goat anti-mouse IgG (H+L) (Thermo Fisher Scientific, A11005) and Alexa Flour 488 goat anti-rabbit IgG (H+L) (Thermo Fisher Scientific, A11008) were used at 1:500. For live confocal imaging of *dat:eGFP* and *dat:mito-RFP* dopaminergic neurons, larvae were mounted in 1.2% low-melting point agarose in E3 embryo medium containing 160 µg/ml ethyl 3-aminobenzoate methanesulfonate (Tricaine MS-222; Sigma-Aldrich 886-86-2) anesthetic. Larva and immunolabeled tissues were imaged on a Zeiss LSM 800 laser scanning confocal microscope. Diencephalic dopaminergic vDC cluster images were acquired by maximum projections of z-stacks of 2 µm sections.

### Quantification and statistical analyses

*dat:eGFP*-positive cell counts in the larval diencephalic dopaminergic neuron dVC cluster were quantified from 12 larvae for each genotype and condition. Quantification was analyzed with two-way ANOVA followed by Tukey's multiple comparison test. RT-qPCR to determine gene expression levels was performed on three independent pools of 30 larvae for each condition. 4C4 microglia counts, mitoRFP/GFP cell counts and RT-qPCR data were analyzed using two-tailed unpaired Student's *t*-test with mean±s.e.m. Gene expression data were analyzed using DeSeq2 (Novogene). Statistical significance was considered at *P*<0.05. Statistical analysis and generation of plots was performed using Prism v.7 software (GraphPad). Western blot band intensities (Fig. S2) were measured using ImageJ and normalized to wild-type band intensity before plotting.

## Supplementary Material

10.1242/dmm.052011_sup1 Supplementary information
