# Supplementary material for: Stat3 mediates Fyn kinase-driven dopaminergic neurodegeneration and microglia activation
Source: Dis Model Mech. 2024 Dec 6;17(12):dmm052011. doi: 10.1242/dmm.052011 (PMC11646115; doi:10.1242/dmm.052011)
Supplement: Supplementary information [file dmm-17-052011-s1.pdf]

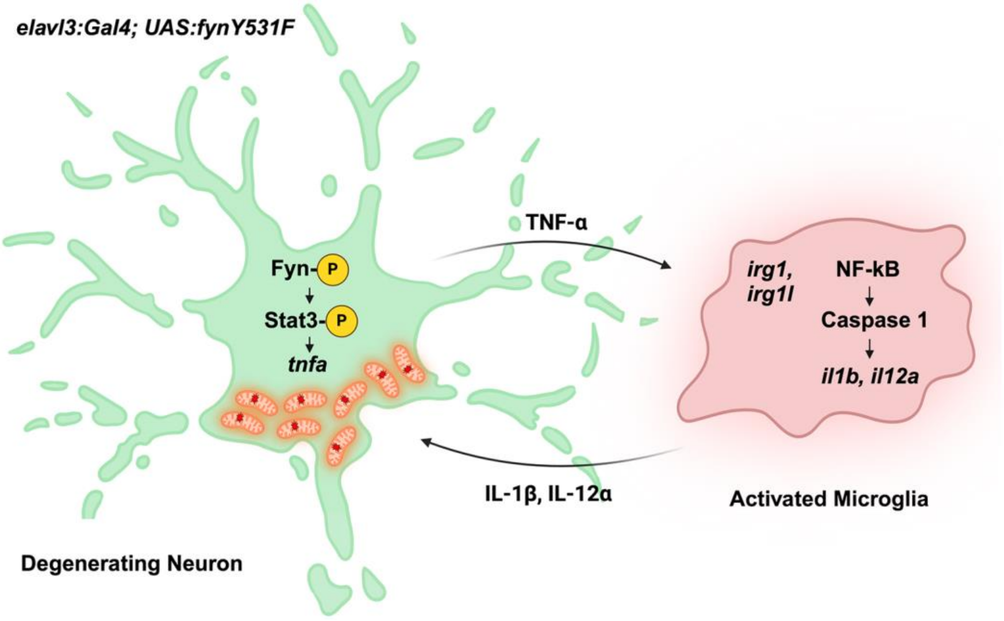

Fig. S1. Summary of findings. Neural Fyn drives dopaminergic neurodegeneration, mitochondria accumulation and microglia activation. Fyn-driven neurodegeneration and cytokine expression are dependent on Stat3. Stat3 and NF-κB pathways synergize in dopaminergic neuron degeneration. This provides a hypothetical model of Fyn-driven cytokine production and feedback loop between neurons and activated microglia.

Disease Models & Mechanisms • Supplementary information

Source data Figure 1 anti-β-actin western

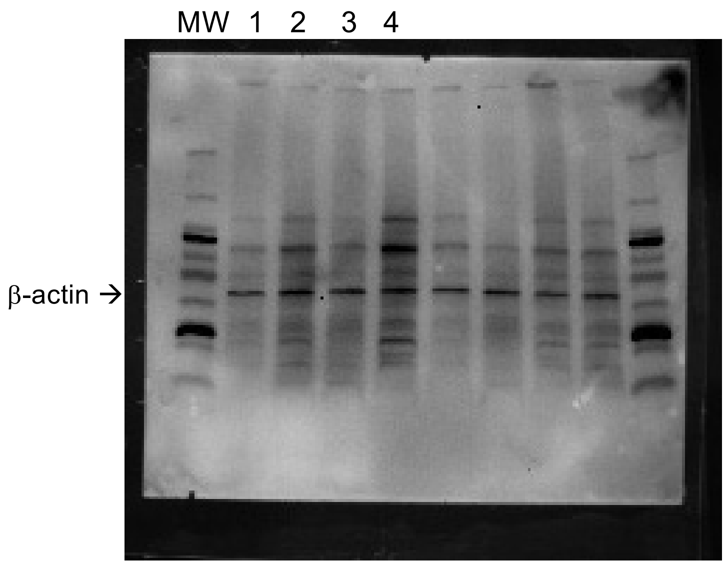

Source data Figure 1 anti-SRC-Y416-PO western

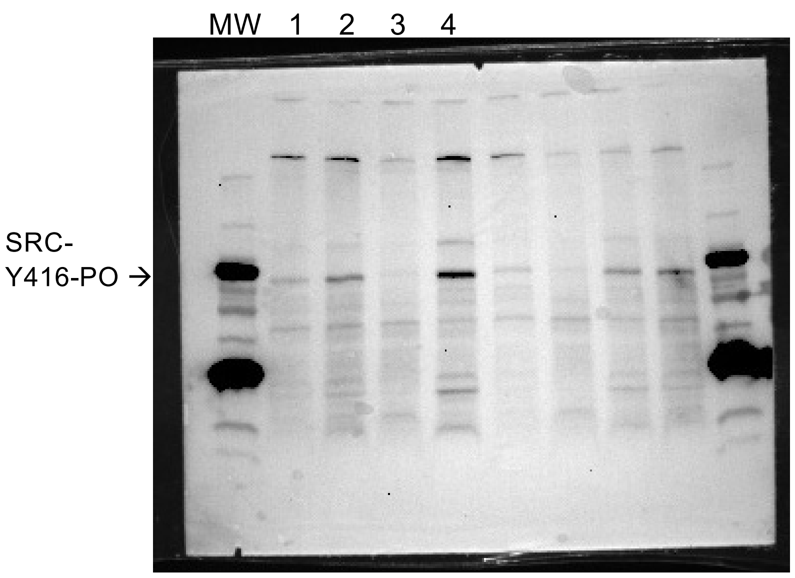

1 +/+
2 elavl3:Gal4; UAS:fynY531Fis89
3 +/+
4 elavl3:Gal4; UAS:fynY531Fis90

Source data Figure 6 anti-β-actin western

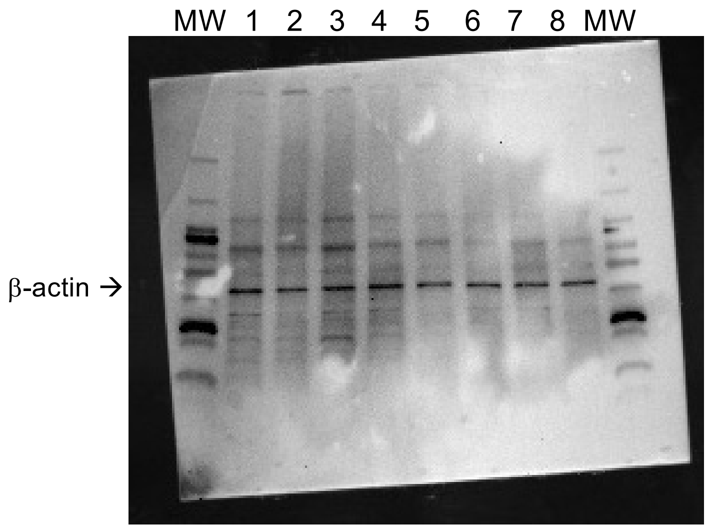

Source data Figure 6 anti-STAT3-Y705-PO western

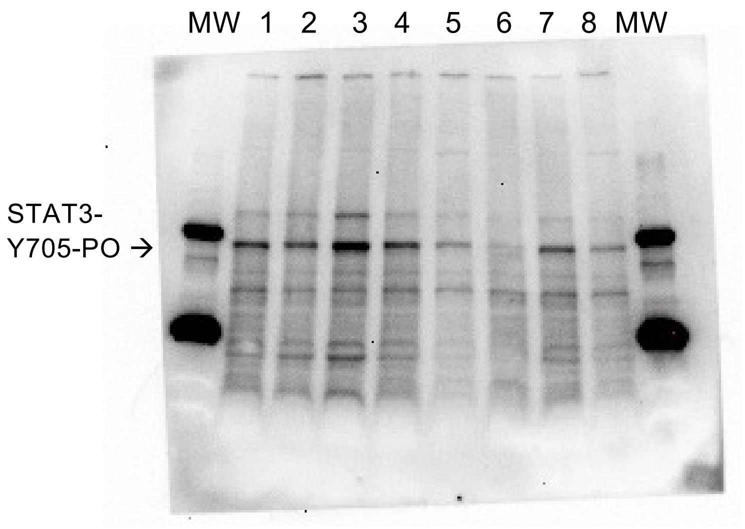

1 +/+
2 elavl3:Gal4
3 elavl3:Gal4; UAS:fynY531F
4 elavl3:Gal4; UAS:fynY531F
5 elavl3:Gal4
6 +/+
7 elavl3:Gal4; UAS:fynY531F + DMSO
8 elavl3:Gal4; UAS:fynY531F + 10μm Saracatinib

Fig. S2. Source data western blots

Disease Models & Mechanisms • Supplementary information

Table S1. Oligonucleotide primers used in this study

Gene	Gene ID	ensembl ID	Forward primer (3′-5′)	Reverse primer (5′-3′)
Fyn	*fyna*	ENSDARG00000011370	GGGGTACCGCCGCCACCATGGGCTGTGTGCAATGTAAG	ATGCGCGCTTAGAGGTTGTCCCCGGGTTG
FynY531F	*fynaY531F*		GGGGTACCGCCGCCACCATGGGCTGTGTGCAATGTAAG	ATGCGCGCTTAGAGGTTGTCCCCGGGTTGGAAC
IL-1β	*il1b*	ENSDARG00000098700	GCATGTCCACATATGCGTC	AGCTGGTCGTATCCGTTTGG
IL-12α	*il12a*	ENSDART00000153685	AACTCCTACAAGCCCAGCAC	ACACTCGGTCGTCAAACGAA
TNF-α	*tnfa*	ENSDARG00000009511	CGCTGGTGATGGTGTCTAGG	TTGTTGATTGCCCTGGGTCT
Acod1/Irg1	*acod1*	ENSDARG00000062788	AGTGATCCGAAGGAGCAAGC	GCACCGCAACACCATTAACA
RpS6Kb	*rps6kb1b*	ENSDARG00000058230	TCCTGATGACTCCACACTGA	GGCGAGGTGAACGGATTT

F *fyna* primer underline=KpnI site.
R *fyna* primer underline=BssHI site.
GAA reverse codon for Y531F mutation.
wildtype *fyna*: G TAC CAA CCC GGG GAC AAC CTC TAA.
fynY531F: G TTC CAA CCC GGG GAC AAC CTC UAA.

Disease Models & Mechanisms • Supplementary information
